# Chronodisruption: Origin, Roots, and Developments of an 18-Year-Old Concept. Comment on Desmet et al. Time-Restricted Feeding in Mice Prevents the Disruption of the Peripheral Circadian Clocks and Its Metabolic Impact during Chronic Jetlag. *Nutrients* 2021, *13*, 3846

**DOI:** 10.3390/nu14020315

**Published:** 2022-01-13

**Authors:** Thomas C. Erren, Claus Piekarski, Russel J. Reiter

**Affiliations:** 1 Institute and Policlinic for Occupational Medicine, Environmental Medicine and Prevention Research, Faculty of Medicine and University Hospital Cologne, University of Cologne, Kerpener Str. 61, D-50938 Cologne, Germany; claus.piekarski@uni-koeln.de; 2 Department of Cell Systems and Anatomy, UT Health San Antonio, San Antonio, TX 78229, USA; REITER@uthscsa.edu

We read with interest the article by Desmet and colleagues entitled “Time-Restricted Feeding in Mice Prevents the Disruption of the Peripheral Circadian Clocks and Its Metabolic Impact during Chronic Jetlag” [1]. A key focus is “chronodisruption”—used some 18 times as “a disruption of the circadian system”—and the authors refer to “chronodisruptors”, for which they include a reference [2]. Since chronodisruption (CD) itself is not explicitly referenced, may we complement their work with publications in which the concept was first proposed in 2003 [3] and systematically developed thereafter?

More generally, CD was conceptualized as a relevant disruption of an otherwise beneficial circadian organization of physiology, endocrinology, metabolism, and behavior by ordered sequences of biological rhythms during sleep and wake cycles. Since the term was coined [3], CD was—step-by-step—put into thematic and historical context with Pittendrigh’s insights as a nestor of modern chronobiology [2,4], included in cancer theory development [5], investigated in shift workers [5,6,7] and flight personnel [6], defined in further detail [2], operationalized as split nexus of internal and external times [8], included in metrics to compute CD doses (Computing chronodisruption—Computing circadian misalignment—Computing sleep deficiency) [7,9,10], contrasted with the concept of social jetlag [9,10], and conceptualized as a ubiquitous causal phenomenon at both work and play [11] (Table 1). Beyond epidemiological contexts, the CD concept is increasingly used, and explored, in experimental research and in more and more journals [12,13].

Clearly, we appreciate the work by Desmet and colleagues. Equally clearly, that CD is a widely used and useful concept is also evinced by citation statistics: as of 3 December 2021, Web of Science indicates that publications that explicitly regard the topic chrondisruption accumulate an h-index of 40 [14].

Overall, that our internal 24 h (circadian) timing systems coordinate countless fundamental physiological processes and that their disruptions may lead to adverse health effects such as obesity [15], diabetes, cardiovascular disease, kidney disease [16], psychiatric disorders, detrimental pregnancy outcomes [17], and, plausibly, cancer [18] can make chronodisruption a prime target for research. An appropriate way to facilitate learning about, challenging, falsifying, or expanding the concept of CD may be to offer source references for its origin [3], roots, and developments (such as in Table 1, [16], and this comment).

## Figures and Tables

**Table 1:** Origin, roots, and developments of the term and concept of chronodisruption.

References	
1960 [4]	Pittendrigh, C.S. Circadian rhythms and the circadian organization of living systems. *Cold Spring Harbor Symposia on Quantitative Biology*
2003 [3]	Erren, T.C.; Reiter, R.J.; Piekarski, C. Light, timing of biological rhythms, and chronodisruption in man. *Naturwissenschaften*
2008 [5]	Erren, T.C.; Reiter, R.J. A generalized theory of carcinogenesis due to chronodisruption. *Neuroendocrinology Letters*
2008 [6]	Erren, T.C.; Pape, H.G.; Reiter, R.J.; Piekarski, C. Chronodisruption and cancer. *Naturwissenschaften*
2009 [2]	Erren, T.C.; Reiter, R.J. Defining chronodisruption. *Journal of Pineal Research*
2013 [8]	Erren, T.C.; Reiter, R.J. Revisiting chronodisruption: when the physiological nexus between internal and external times splits in humans. *Naturwissenschaften*
2014 [7]	Erren, T.C.; Morfeld, P. Computing chronodisruption: how to avoid potential chronobiological errors in epidemiological studies of shift work and cancer. *Chronobiology International*
2017 [9]	Erren, T.C.; Morfeld, P.; Lewis, P. Computing circadian misalignment: Why not combine sleep timing and duration to assess accumulated sleep deficiency? *Chronobiology International*
2018 [10]	Erren, T.C.; Gross, J.V.; Lewis, P. Computing sleep deficiency. *Journal of Sleep Research*
2019 [11]	Erren, T.C.; Lewis, P. Hypothesis: ubiquitous circadian disruption can cause cancer. *European Journal of Epidemiology*
